# Juvenile dermatomyositis without skin lesions

**Published:** 2015-07-06

**Authors:** Yalda Nilipour, Maryam Ghiasi, Mohammad Rohani, Fatemeh Omrani

**Affiliations:** 1 Pediatric Pathology Research Center, Mofid Children Hospital, Shahid Beheshti University of Medical Sciences, Tehran, Iran; 2 Department of Dermatology, Razi Hospital, Tehran University of Medical Sciences, Tehran, Iran; 3 Department of Neurology, Rasool Akram Hospital, Iran University of Medical Sciences, Tehran, Iran; 4 Rasool Akram Hospital, Iran University of Medical Sciences, Tehran, Iran

**Keywords:** Juvenile Dermatomyositis, Skin Lesions, Iran

## Introduction

An 8-year-old Iranian girl was referred because she had progressive muscle weakness predominantly in lower limbs since about 2 years ago. She was not able to stand from a sitting position without help and had difficulty climbing stairs. She walked slowly and could not run like before. She had no complaint of dysphagia or dysphonia.

She was born through a normal vaginal delivery and had a history of neonatal jaundice treated with phototherapy. She was taking no medications and had no history of cutaneous disease or photosensitivity. Her parents mentioned no recent weight loss.

Family history was negative for neuromuscular disorders. Her parents were not related.

On physical examination, the patient was an alert young girl with stable vital signs; Oral temperature: 36.8, Heart rate: 88 beats/min, respiratory rate: 22/min, and blood pressure 115/70 mm Hg. Her weight was 23 kg. She had mild lumbar lordosis without pes cavus, no kyphoscoliosis or other musculoskeletal deformities. She had waddling gait with positive Gower’s sign. She was able to walk on heel and toe and had mild atrophy of hamstring muscles. There was no muscle tenderness.

She had no facial weakness and no dysphonia. Her muscle forces were as: neck flexion 4/5, neck extension 4/5, proximal upper limbs 4/5, proximal lower limbs 3+/5, foot dorsiflexion, and plantar flexion were normal.

Skin examination by an expert dermatologist showed no abnormalities on the face, hands or fingers.

Ancillary investigations showed: Serum creatine kinase activity as 78. Serum aldolase level was also normal. Aspartate aminotransferase was 37 and Alanine aminotransferase was 19. Fluorescent antinuclear antibody (FANA), anti-neutrophil cytoplasmic antibody, anti–double-stranded DNA antibodies, and rheumatoid factor were all negative. Thyroid function tests, complete blood count, and urine analysis were also normal.

Cardiological investigations were normal. Nerve conduction studies in upper and lower limbs were normal [including low-frequency and high-frequency repetitive nerve stimulation (RNS)]; but on needle examination all of the tested muscles in lower and upper limbs [deltoid], first dorsal interosseous (FDI), gluteus medius and maximus, rectus femoris, anterior, and gastrocnemius revealed typical myopathic pattern [small polyphasic motor unit action potentials (MUAPs) with early recruitment] without spontaneous activity [there was no fibrillation, positive sharp wave (PSW), myotonia or fasciculation].

**Figure 1:**
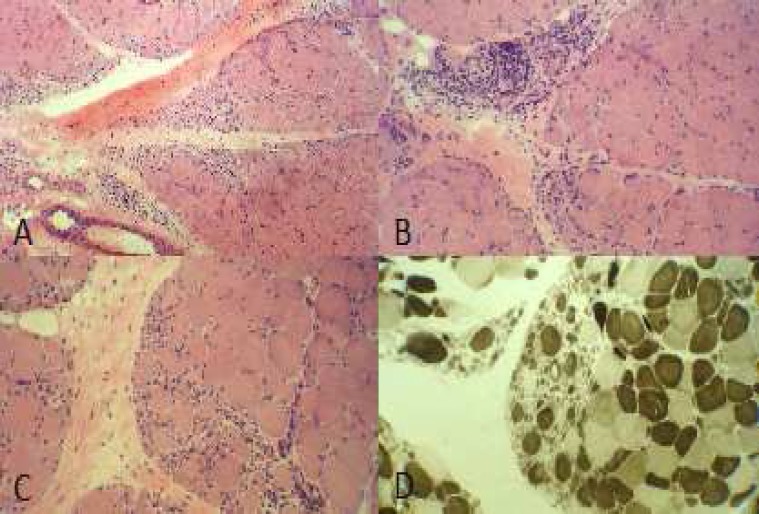
(a) Prominent fiber size variation with atrophy and degeneration/regeneration of the fibers exclusively arranged in the periphery of the fascicles (H and E, ×40). (b) Perimysial perivascular infiltration of chronic inflammatory cells with perifascicular degenerative/regenerative fibers and increased internalized nuclei (H and E, ×200). (c) Group atrophy with the typical perifascicular pattern (H and E, ×200). (d) Checkerboard pattern with no fiber type grouping (ATPase PH 4.63, ×200)

She was referred for muscle biopsy and muscle biopsy from her left deltoid muscle reveal prominent typical perifascicular atrophy pattern in many fascicles (Figure 1a, 1C) with some foci of perimysial perivascular chronic inflammatory cell infiltration (Figure 1b). ATPase study revealed no fiber type grouping and atrophic fibers were both type 1 and 2. The diagnosis of dermatomyositis was made based on typical pathognomonic findings of her muscle biopsy.

The patient received methylprednisolone pulse (500 mg/day for 5 days), the muscle forces mildly improved and she was discharged with oral prednisolone (1 mg/kg/day).

On follow-up visit, 1-month later, she showed good response to treatment and her muscle forces had been improved significantly and she was able to run and stand without difficulty from sitting position but she had mild lumbar lordosis yet. 

Idiopathic inflammatory myopathies are a group of disorders including dermatomyositis, polymyositis, autoimmune necrotizing myopathy and inclusion body myositis. Although polymyositis is rare in children, but juvenile dermatomyositis (JDM) is more frequent which is characterized by disease onset under the age 16.^1^ Dermatomyositis is more common in females (female/male ratio is 2:1), but in juvenile DM males and females are equally involved (the F/M ratio is about 1:1).^2^ Historically, dermatomyositis had been differentiated from polymyositis only by dermatologic features, but they are now known as two different diseases with different pathophysiology, pathology, and clinical courses. Perifascicular atrophy is a particular feature of dermatomyositis that is not seen in polymyositis.^3^ DM is characterized by infiltration of inflammatory cells in muscle and skin capillaries and perifascicular inflammation and atrophy.

In a retrospective study of 166 patients with JDM, children with untreated JDM were shorter and lighter than national norms which indicate the importance of the diagnosis and treatment of JDM.^2^

Most of the DM patients have both symptoms of myopathy and cutaneous involvement. Some patients have only dermatologic manifestations and are named “amyopathic dermatomyositis.” Skin lesions usually precede muscle weakness but sometimes they may occur at the same time or even after myopathy.^1^^,^^4^ Very occasionally patients have no skin rash, but the muscle biopsy shows dermatomyositis. These patients are called “dermatomyositis sine dermatitis.” In this group, muscle biopsy leads to a correct diagnosis.^1^^,^^5^

In our patient, cutaneous manifestations may occur later (although we did not see cutaneous manifestations in our patient after 4 months follow-up).

This makes the role of muscle biopsy more important in diagnosis of inflammatory muscle diseases, since clinical features cannot always differentiate between subtypes of inflammatory myopathies or between inflammatory myopathies and hereditary myopathies such as muscular dystrophies or metabolic myopathies.

## Conflict of Interests

The authors declare no conflict of interest in this study.

## Notes:

**How to cite this article: **Nilipour Y, Ghiasi M, Rohani M, Omrani F. Juvenile dermatomyositis without skin lesions. Iran J Neurol 2015; 14(3): 171-3.
